# A Rare Case of Cardiac Metastasis from Colon Cancer

**DOI:** 10.30699/IJP.2022.551822.2874

**Published:** 2022-08-02

**Authors:** Zahra Modabber, Roghayeh Pourkia, Hamidreza Vafaey, Ali Alizadeh, Mohammad Ranaee

**Affiliations:** 1 *Student Committee Research, Clinical Research Development Unit of Rouhani Hospital, Babol University of Medical Sciences, Babol, Iran*; 2 *Clinical Research Development Unit of Rouhani Hospital, Babol University of Medical Sciences, Babol, Iran*; 3 *Cellular and Molecular Biology Research Center, Health Research Institute, Clinical Research Development Unit of Rouhani Hospital, Babol University of Medical Sciences, Babol, Iran*

**Keywords:** Cardiac metastasis, Colorectal neoplasms, Heart, Left atrium, Metastatic adenocarcinoma

## Abstract

It is very rare for colorectal neoplasms to metastasize to the heart in the worldwide medical literature; only a single case of well-documented colorectal cancer metastasis to the left atrium was found. The case of a 66-year-old man is explained in this paper, who was suffering from metastatic adenocarcinoma of the colon that included the left atrium. In transthoracic and transesophageal echocardiography, a large multilobulated mass was present in the left atrium. An accidental pulmonary mass was also seen in a lung spiral CT scan. The cardiac mass was taken out, and a biopsy was obtained from the pulmonary mass. Adenocarcinoma was seen in histological assessment. Immunohistochemical staining was carried out to examine the expression of cytokeratin 7, cytokeratin 20, and caudal-related homeobox transcription factor 2 (CDX2) to determine the origin of the adenocarcinoma. In addition, the expression of these proteins was linked to the attributes of the patient and tumor. Post-surgical transesophageal echocardiography showed normal left ventricle and right ventricle function with no evidence of left atrium mass. Therefore, we suggest that asymptomatic cancer patients with a history of colorectal cancer and who have developed cardiac symptoms should be immediately examined for potential cardiac metastasis.

## Introduction

Colorectal cancer (CRC) is the third most widely identified and the second most lethal cancer in males as well as females from all over the world (1). The following are the modifiable factors for CRC: alcohol consumption, tobacco exposure, insufficient diet, and obesity; these factors are the main reasons the incidence and mortality of CRC have increased (2). Metastases from CRC can either occur through lymphatic or hematogenous spreading, and the liver and the lungs are the most common sites involved (3, 4). It has been found that the spleen, spermatic cord, thyroid gland, and skeletal muscles are some of the organs where uncommon metastases occur from colorectal cancer (5, 6). Cardiac metastasis rarely occurs from CRC, and its incidence is likely underestimated (7). Though very few cases of clinical diagnosis of cardiac metastasis from CRC persistently serve as a diagnostic and therapeutic challenge, it is important to consider this neoplasm as part of the wide differential of cardiac intracavitary masses (7). It was found in an extensive autopsy series that colon carcinoma metastases to the heart and constitutes 1.2% of all metastatic neoplasm, having a prevalence of 2% (8). A few cases of cardiac metastasis due to colorectal cancer have been explained earlier. In this study, we presented a case of CRC and metastasis over the left atrium (LA) that presented with transient ischaemic attack (TIA).

##  Case Report

**Fig. 1:**
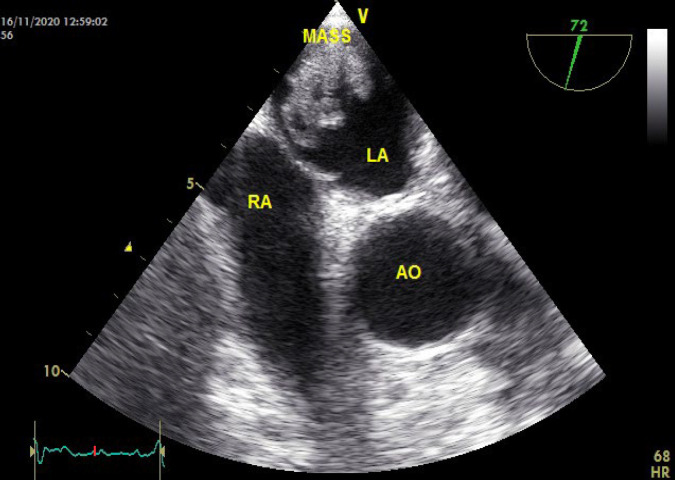
A large size mass (4×2.5cm) in the left atrium protruding into the left upper pulmonary vein shown in the short axis transesophageal echocardiogram view

**Fig. 2:**
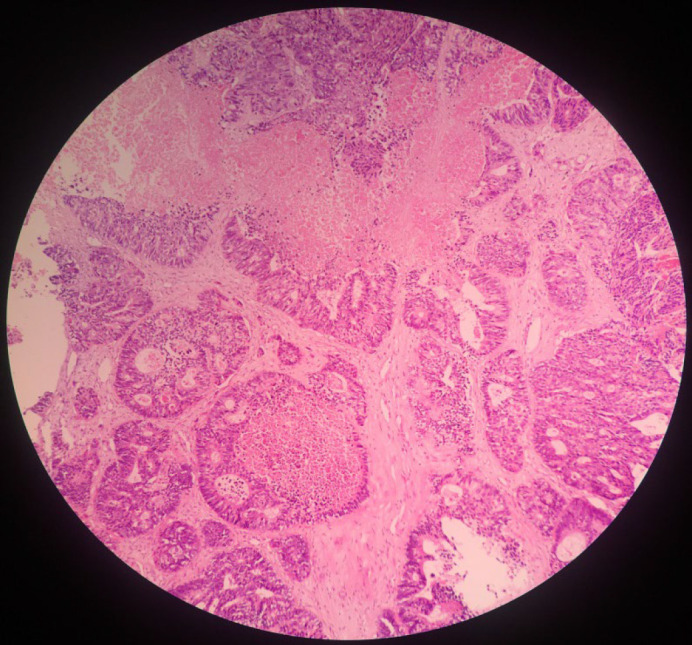
Photomicrograph of the heart mass showing a moderately differentiated adenocarcinoma. (H&E stain)

**Fig. 3:**
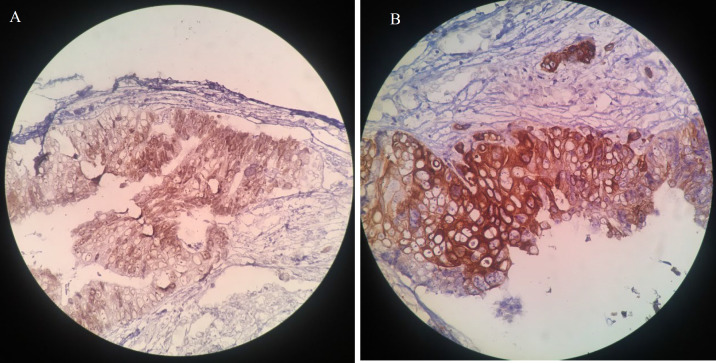
Photomicrograph showing a diffuse positivity for CK20 (A) and CDX2 (B). (IHC stain)

A 66-year-old male was admitted to our hospital in late 2020 as he was experiencing transient dysarthria. The patient had undergone right hemicolectomy and adjuvant chemoradiation for adenocarcinoma 3 years back. His medical history included a cerebrovascular accident in the past and recent depression. The patient's social history was not unusual. The laboratory findings at the time of admission for complete blood count (CBC), coagulation tests, and liver function tests were within the given limits. There was an increase in erythrocyte sedimentation rate (ESR) and C-reactive protein (30 and 48, respectively). A positive result was obtained for the serological test for hepatitis B. Physical examination did not show any sign, and the jugular venous pulse was also normal. Neurological examination and brain CT scan were unremarkable. The patient was diagnosed with a transient ischemic attack (TIA). Transthoracic echocardiography (TTE) was requested to investigate the origin of the embolic lesion of the brain. It showed LA mass, normal LV and RV function, normal pulmonary artery pressure (PAP), and no significant valvular heart disease (VHD). Transesophageal echocardiography (TEE) was performed to examine the LA mass more closely. A large size (4×2.5cm), mobile, hypochondriac, multilobulated mass was seen in the LA roof at the right upper pulmonary vein entrance site with protrusion into it (Figure 1). An accidental pulmonary mass was also seen in a lung spiral CT scan. The cardiac mass was taken out to prevent embolism, and a sample for biopsy was obtained from the pulmonary mass. The mass was friable and creamy. The histopathologic assessment showed atypical glandular proliferation with cribriform structures lined by neoplastic cells with nuclear hyperchromasia and pleomorphism and was diagnosed as metastatic moderately differentiated adenocarcinoma (Figure 2). The expression of cytokeratin 7 (CK7), cytokeratin 20 (CK20), and caudal-related homeobox transcription factor 2 (CDX2) was examined using immuno-histochemical staining so that the origin of the adenocarcinoma could be verified (Figure 3). The expression of these proteins was related to the attributes of the patient and tumor.

TTE after surgery and follow-up did not show any mass in the heart until nine months later. The patient had no signs of embolism during this time.

## Discussion

CRC is the second or third most widely prevalent human malignancy and the leading cause of cancer-related deaths (9, 10).

Autopsy series of cancer patients have shown that the prevalence of metastatic cardiac tumors is between 1.5% to 20% (11). It is very rare to observe metastasis of CRC to the heart (autopsy series have shown that the prevalence is 1.4% to 2% compared to 23% to 31% for bronchogenic carcinomas), and it usually involves the pericardium (12). Typically, cardiac metastasis is asymptomatic, and its occurrence is underestimated (13).

A few cases of well-documented metastatic CRC concerning the right atrium or ventricle were obtained from the international medical literature (13-16). 

LA involvement was explained by Reisenauer* et al.* as a consequence of tumor extension from rectal adenocarcinoma (17). According to the available information, this is the second case report that explains LA involvement due to an extension of the tumor through colon metastasis.

CK7 and CK20 are cytokeratins with low molecular weight. Several primary and metastatic carcinomas have been reviewed to examine the expressions of CK7 and CK20 (18). The past few studies have focused on identifying the origin of metastatic carcinomas through CK7 and CK20 (19-21).

CDX2, an intestine-specific transcription factor, is a highly specific and sensitive marker of intestinal differentiation (22). Loss of CDX2 reflects weak response and low survival benefits from standard chemotherapy in case of metastasis (23).

Two recent retrospective studies have found that CDX2 loss functions as a predictive biomarker for the effectiveness of chemotherapy treatment in stages II and III of CRC (24, 25). 

The patient did not exhibit any major sign base when physically examined, and the expression of CK7, CK20, and CDX2 was examined to determine the origin of his tumor.

The infrequency of cardiac metastasis may be because of the following factors: the powerful kneading action of the myocardium, the rapid movement of blood through the heart, metabolic peculiarities of striated muscle, and the normal movement of lymph flow away from the heart (13). Quite often, the right side of the heart is involved rather than the left side (7). Cardiac metastases are clinically manifested as protean and are determined by the metastatic tumor's position and size. However, there may be more extensive involvement with little or no symptoms, and over 90% of the time, metastatic cardiac tumors are clinically silent (11, 26). 

Metastasis in the heart can be diagnosed by performing echocardiography (27). However, it is possible to achieve false positive and false negative findings (27). This method is non-invasive and can examine the physiological and anatomic features of the heart (27). To diagnose cardiac metastases, transthoracic echocardiography (TTE) can be used (7). In this study, TTE was also used and showed this mass.

 It was shown in Patel* et al.*'s study that TEE showed a lobulated, multicystic mass with an evident delineated pedicle connected with the RA-free wall (7). A large mass that occupies most of the LA is depicted by TEE in Reisenauer* et al.* (17), which may possibly hamper the inflow to the LV (28). TEE, in this case report, showed a large, mobile, multilobulated mass measuring 4 x 2.5 cm in the left atrium.

When cardiac tumors are definitively diagnosed, they have a weak prognosis. Chemotherapy, radiation, or resection may be appropriate in case the metastasis is solitary; however, it is vital to consider metastatic burden and illness volume, which frequently determines the treatment modality (29). The surgery's role in heart metastasis is not adequately established. Surgery is advised when there is an obstruction (10).

Surgery may improve quality of life and survival, but only in a few selected cases (10). According to Koizumi* et al.* (28), though surgery is advised in very rare cases as a treatment for metastatic cardiac tumors, surgical treatment may particularly be effective in cases of obstructive and solitary lesions to provide relief from symptoms and an extension of life expectancy. The goal of surgical treatment in our case was to prevent mass embolism again.

A case of heart metastasis was identified by Tsujii, with over two-year survival and only chemotherapy without surgery (9). It is not always possible to carry out the surgery.

In this context, a case of heart metastasis was presented by Nishida, where the patient passed away two weeks following the tumor resection (30).

A 68.4% rate of survival was shown by Murphy and colleagues (31) in 19 patients who had undergone resection for metastatic cardiac disease for different reasons over a span of 25 years. Hence, an aggressive surgical approach was suggested by the authors.

In another case, a patient had to undergo right atrial colorectal metastasis resection with bovine pericardial patch reconstruction. The patient suffered from bleeding on the third postoperative day and passed away (13).

LA metastases were fully removed by Reisenauer* et al.* from colon cancer in a 65-year-old man. The surgery was effectively carried out on our patient, and following the surgery, the TTE showed that the patient had normal size and function of the left ventricle (17).

Hence, additional studies are required to separate the role of surgical treatment in cardiac metastasis from colorectal cancer.

Another option is chemotherapy, which was also performed on our patient; however, it is often of a palliative nature. No standardized methods have been determined till now for treating patients with cardiac metastasis from colorectal cancer. Therefore, additional studies need to be carried out to determine the most appropriate treatment for these patients.

## Conclusion

We reported metastatic involvement of the left atrium from colorectal cancert which is rare. A large mobile mass was seen in transthoracic echocardiogram (TTE), which induced pressure on the right ventricle and atrium. A multilobulated mass was shown by TEE in the left atrium, measuring 4 x 2.5 cm, in addition to accidental pulmonary mass. Following the cardiac biopsy, Immunohistochemical staining for CK7, CK20, and CDX2 was performed to confirm the presence of adenocarcinoma. It was shown by TTE examination that the surgery was effective and the size and function of the left ventricle were found in normal limit.

In conclusion, surgery may be appropriate treatment for given patients with metastatic cardiac tumors; however, no standardized methods have been established yet for treatment of the patients with cardiac metastases from CRC. Additional studies should be conducted to determine the most effective treatment for these patients.

##  Ethics Approval & Consent to Participate

Not Applicable.

##  Authors' Contributions

Not Applicable. 

## Conflict of Interest

The authors declared no conflict of interest.

## Funding

The author(s) received no financial support for the research, authorship, and/or publication of this article.
